# Low Prevalence of Hepatitis C Virus Infection Among HIV-Positive Patients: Data From a Large-Scale Cohort Study in Istanbul, Turkey

**DOI:** 10.5812/hepatmon.18128

**Published:** 2014-08-16

**Authors:** Ozlem Altuntas Aydin, Mucahit Yemisen, Hayat Kumbasar Karaosmanoglu, Fatma Sargin, Alper Gunduz, Bahadir Ceylan, Bilgul Mete, Nail Ozgunes, Dilek Yildiz Sevgi, Resat Ozaras, Fehmi Tabak

**Affiliations:** 1 Department of Infectious Diseases and Clinical Microbiology, Haseki Training and Research Hospital, Istanbul, Turkey; 2 Department of Infectious Diseases and Clinical Microbiology, Cerrahpasa Medical Faculty, Istanbul University, Istanbul, Turkey; 3 Department of Infectious Diseases and Clinical Microbiology, Goztepe Training and Research Hospital, Istanbul Medeniyet University, Istanbul, Turkey; 4 Department of Infectious Diseases and Clinical Microbiology, Sisli Etfal Training and Research Hospital, Istanbul, Turkey; 5 Department of Infectious Diseases and Clinical Microbiology, Medical Faculty, Bezmialem Vakif University, Istanbul, Turkey

**Keywords:** Human Immunodeficiency Virus, Hepatitis C Virus, Prevalence, Turkey

## Abstract

**Background:**

Rate of coinfection with human immunodeficiency virus (HIV) and hepatitis C virus (HCV) varies in different countries. This may be attributable to common transmission routes as well as social, economic, and cultural factors.

**Objectives:**

The purpose of this study was to investigate the prevalence and risk factors of HCV infection among HIV-positive patients in Istanbul, Turkey.

**Patients and Methods:**

Since January 2006 to November 2013, 949 HIV-positive patients that were enrolled in this study by ACTHIV-IST (Action Against HIV in Istanbul) Study Group, which consists of five centers to follow up HIV-positive patients in Istanbul. Epidemiologic and clinical data were collected retrospectively from medical records and were transferred to an HIV database system.

**Results:**

Among 949 patients, 84% were men and the mean age was 37.92 ± 11.54 years (range, 17-79). The most frequent route of transmission was heterosexual intercourse (48.8%), followed by men having sex with men (30.5%). Only nine patients (0.9%) had history of injection drug use (IDU). The prevalence of HIV/HCV coinfection was 0.9% (9:949). The IDU rate was 44.4% (4:9) in patients with HIV/HCV coinfection (three of them were not Turkish citizens), whereas this rate was only 0.6% (5:881) in patients with only HIV infection (P < 0.01). Genotypes 1b, 2a/2c, and 3 were determined in five, one, and two patients, respectively. Genotype could not be determined in one patient. History of residence in a foreign country (P < 0.01) and imprisonment (P < 0.01) were also considered as risk factors in terms of HIV/HCV coinfection.

**Conclusions:**

Prevalence of HIV/HCV coinfection is considerably low in Turkey. The extremely rare prevalence of IDU might have a role in this low prevalence.

## 1. Background

Coinfection with immunodeficiency virus (HIV) and hepatitis C virus (HCV) is commonly observed due to their common routes of transmission. Moreover, almost one-quarter of patients with HIV/AIDS are also infected with HCV (1). In areas where highly active antiretroviral therapy (HAART) is available, HCV infection has become a major cause of mortality among HIV-positive patients and hence, the life expectancy has decreased (2). Patients with HIV/HCV coinfection are more likely to develop cirrhosis and have an increased risk of developing AIDS (3). For these reasons, American and European guidelines recommend HCV screening of all HIV-positive individuals (4-6).

The rate of HIV/HCV coinfection differs from country to country. This may be attributable to their common transmission routes and social, economic, and cultural factors. For HIV and HCV, the most common transmission routs are exposure to blood and injections drug use (IDU). In the developed countries such as the United States, Australia, and many European countries, IDU is the dominant route of HCV transmission. However, the transmission is attributed to unsafe therapeutic injections for HCV infection in Egypt (with the highest reported HCV seroprevalence worldwide) and in India (7). The main route of HIV transmission differs by region. For instance, the main route is heterosexual intercourse in sub-Saharan Africa and homosexual intercourse in North America and Latin America. Nevertheless, IDU is the most common route of HIV transmission in Eastern Europe and Central Asia. Although heterosexual transmission accounts for the largest proportion of HIV transmission in Western Europe, the incidence has increased in men who have sex with men (MSM) during recent years (8). It has been reported that in Middle Eastern countries such as Iran, IDU is the most common high-risk behavior for HIV and HCV transmission (9). The risk of vertical transmission is high for HIV and relatively low for HCV, but the risk increases for HIV-positive mothers (10). Although rate of HCV transmission by sexual route is low, incremental trend of detecting HCV among HIV-positive MSM has been reported from many countries recently (11, 12). 

Turkey is among low-prevalence countries in Europe in terms of both HIV and HCV infections. Despite common transmission routes, data on HCV seroprevalence among patients with HIV/AIDS in our country is insufficient.

## 2. Objectives

The aim of this study was to determine the prevalence and associated risk factors of HCV infection among HIV-positive patients who were followed up by five centers in Istanbul, a large cosmopolitan city in Western Turkey.

## 3. Patients and Methods

The HIV-positive patients were enrolled in this retrospective study by ACTHIV-IST (Action against HIV in Istanbul) Study Group, which consists of five centers to follow up HIV-positive patients in Istanbul. Three of these centers are located in university hospitals and two are in public training hospitals. All newly diagnosed HIV/AIDS patients with confirmed diagnosis through Western Blot verification test (HIV BLOT 2.2, MP Biomedicals Asia Pacific, Singapore) who attended the abovementioned clinics between January 2006 and November 2013 were included. None of the patients with HIV/HCV coinfection had received treatment for HCV. The patients were screened for HCV antibodies by ELISA method (Inno-test HCV Ab IV, Innogenetics, Belgium) and HCV-RNA levels of seropositive patients were detected by polymerase chain reaction (PCR) (COBAS Ampliprep/COBAS TaqMan 96, Roche Molecular Systems, USA). Sera with detected HCV RNA were genotyped (Abbott RealTime HCV Genotype II, IL). The CD4+ cell counts were obtained by standard flow cytometry (FACScalibur, Becton Dickinson, New Jersey, USA) and HIV viral load was measured by PCR (COBAS Ampliprep/COBAS TaqMan HIV-1 Test, Roche Molecular Systems, USA). Demographic data including age, sex, transmission routes, education level, marital status, and history of imprisonment, CD4+ counts, HIV RNA, Anti-HCV, and HCV RNA were collected retrospectively from medical records and were transferred to a HIV database system. 

All analysis were performed by using GraphPad Prism 5.0 (GraphPad Software, Inc., San Diego, CA, USA) and SPSS 15 (SPSS Inc, Chicago, IL, USA). Data were described using mean ± standard deviation (SD) (or median and range) and as an absolute number and percentage when indicated. The student t test was used to analyze quantitative data. A P value < 0.05 was considered as statistically significant.

## 4. Results

A total of 949 naive HIV-positive patients were enrolled in this study. Among the included patients, 797 (84%) were male and the mean age was 37.92 ± 11.54 years (range, 17-79). Moreover, 21 patients (2.2%) were not Turkish citizens. Most frequent route of HIV transmission was heterosexual intercourse (48.8%) and MSM (30.5%), consecutively. Only nine patients (0.9%) had history of injection drug use (IDU). The CD4+ lymphocyte count and HIV RNA had been performed on 895 of the cases. Mean CD4+ lymphocyte count at the first admission was 364.24 ± 280.83/mm3 (range, 0-2106) and mean plasma HIV RNA was 4.97 ± 0.97 log10 copies/mL. The CD4+ lymphocyte count < 200/mm³ and > 500/mm3 were reported in 223 (24.9%) and 220 patients (24.6%), respectively.

The number of HIV-positive individuals with positive titers for anti-HCV antibodies was 9 (0.9%), consisting of five females and four males, with detection of HCV RNA in all of them. The hepatitis B virus surface antigen (HBsAg) was detected in 59 patients. Nevertheless, the HCV/HBV/HIV coinfection was not identified in any patients. Table 1 shows the demographic characteristics of the patients according to HCV seropositivity (Table 1). In patients with HIV/HCV coinfection, the IDU as the route of HIV transmission, residence in a foreign country, and history of imprisonment had a significant association with HIV/HCV coinfection. 

**Table 1.** Demographic Characteristics of Patients According to Hepatitis C Virus Seropositivity

	Patients With HIV/HCV Coinfected, (n = 9), No. (%)	HIV-Positive Patients, (n = 940), No. (%)	P value
**Mean Age, y**	34	38	0.42
**HIV Transmission Route**			< 0.01
Heterosexual	5 (55.5)	466 (49.6)	
Homosexual	0	289 (30.7)	
Intravenous Drug Use	4 (44.5)	5 (0.5)	
**Level of Education, (N = 621)**			0.94
Illiterate	0	15	
Elementary-Secondary	3	231	
Highschool	3	158	
University	3	227	
**Residence in a Foreign Country**			< 0.01
Yes	6 (66.6)	132 (14)	
No	3 (33.4)	808 (86)	
**History of Imprisonment**			< 0.01
Yes	1 (11.1)	10 (1.1)	
No	8 (88.9)	930 (98.9)	

All of the patients with HIV/HCV coinfection were Caucasian. Three of them were not Turkish citizens (two from Moldova and one from Turkmenistan). 

Genotype analysis revealed the genotype 1b, 2a/2c, and 3 in five, one, and two patients, respectively. Genotype could not be determined in one patient.

The mean CD4+ counts for patients with HIV/HCV coinfection and HIV-positive patients were 128 ± 139 and 336 ± 226 cells/mm3, respectively (P = 0.06). No significant difference was detected in terms of HIV-RNA level between two groups (P = 0.924).

## 5. Discussion

This study indicated that the prevalence of HIV/HCV coinfection in our country is considerably low (0.9%). Although occult HCV infection is a concern in HIV-positive patients, HCV-RNA testing is recommended in case of unexplained transaminase elevation in those with a CD4+ < 200/mm³, when acute HCV is suspected, or among subjects with a high risk of acquiring HCV (13). In our study, 223 patients with HIV/HCV coinfection had CD4+ < 200/mm³ and none had high ALT or suspicion of acute HCV infection. Therefore, we did not test HCV RNA in this group.

Depending on the mode of HIV transmission, the prevalence of HIV/HCV coinfection varies from one country to another (14). As the main route of HCV spread is IDU, HIV/HCV coinfection rates are often more than 90% among patients with HIV/AIDS who are injection drug users. High HIV/HCV coinfection rates (around 70%) can be found in Eastern European countries (e.g. Belarus and Ukraine) and in Middle Eastern countries such as Iran where IDU is the main route of HIV transmission (15-17). Nevertheless, in Western Europe, for example in Barcelona, the prevalence of HCV coinfection among newly diagnosed HIV-positive patients had decreased from 24% between 2000-2002 to 10% in the years 2006-2008 as a result of needle exchange programs among injection drug users (18). On the contrary, HCV coinfection among HIV-positive patients was as low as 3.39% in Northeast Brazil, which was associated with low IDU rate in HIV-positive patients (19). 

In Turkey, the first case of HIV/AIDS was reported in 1985. According to the survey conducted by the Turkish Ministry of Health, there were 6854 cases of HIV infection out of a population of 76.6 million people in Turkey between October 1985 and June 2013. In a recent study on the antiretroviral-naive Turkish patients with HIV-1, the transmission route was reported as sexual intercourse (heterosexual contact in 60% and homosexual contact in 38%) and IDU in just 2% (20, 21). According to a study covering the whole country (TURKHEP Study), the HCV seroprevalence rate was 1% in Turkey (22). Moreover, referring to a conducted meta-analysis in our country, while the HCV seroprevalence rate was 0.1% to 0.8% according to the geographic areas, the rates among blood donors, preoperative patients, sex workers, and hemodialysis patients were 0.3%, 0.6% to 1.3%, 2.2%, and 20.4%, respectively (23). In another study by Karaca et al., the most common risk factors of HCV infection were history of surgery (98%), blood transfusion (39.7%), and dental procedure (27.5%). In addition, history of IDU (3.1%) and suspicious sexual intercourse (1.5%) were determined as minor risk factors (24). 

According to the aforementioned studies, heterosexual intercourse is the most frequenr route of HIV transmission in Turkey. Moreover, IDU is the least common risk factor in the transmission of both HIV and HCV. According to a study conducted in Turkey, the IDU prevalence rate had been 0.05% in general population (25). In our study, only 9 patients (0.9%) had the history of IDU among all the cases while the rate was 44.4% (4:9) in patients with HIV/HCV coinfection; however, three were not Turkish citizens and the other three had resided out of Turkey. In addition to IDU, a history of residence in a foreign country was determined as a risk factor in terms of HIV/HCV coinfection in our patients. 

Although rate of sexual transmission of HCV is known to be low in serodiscordant heterosexual couples, there has been a dramatic rise in the incidence of HCV among HIV-infected MSM in Europe, the United States, Australia, and Asia since 2000 (26-29). Increases in HCV seroconversion among HIV-positive MSM have been reported in association with unprotected anal intercourse, multiple sexual partners, rough sexual techniques, and coinfection with HIV and other sexually transmitted infections (2, 14). In our study on the contrary, none of the coinfected patients had reported homosexual intercourse.

Previous studies have revealed at least six major genotypes and more than 70 subtypes of HCV (30). HCV genotypes 1, 2, and 3 have a worldwide distribution, genotype 4 is prevalent in Africa and Middle East, genotype 5 and 6 are found in South Africa and Southeast Asia, respectively (31). According to a multicenter study in Turkey during 2009, HCV genotype 1 was the predominant one (87.5%) in 834 patients (32). Similarly, HCV genotype 1b was also predominant in our study (5:8, 62.5%). Nevertheless, two patients with genotype 3 had been infected in Germany and in China through IDU. The only patient with genotype 2a/2c was infected through IDU in Moldova. 

The possible limitation of this study might be due to the low number of the patients with HIV/HCV coinfection; however, it was unavoidable since the total number of patients with HIV/AIDS coinfection is low in Turkey.

In conclusion, the prevalence rate of HCV among HIV-positive patients in our study was similar to the rate in Turkey. Furthermore, the prevalence level detected in our patients with HIV/HCV coinfection was the lowest ever reported rate in the literature. The extremely rare prevalence of HCV infection among HIV-positive population in our country might be a consequence of the small number of the injection drug users. Additionally, relatively low rate of MSM among our HIV-positive patients might have a role in low HIV/HCV coinfection prevalence. Although the prevalence of HCV coinfection is low among HIV-positive patients, HCV should also be screened in all patients with HIV/AIDS. 
